# Self-Assembled Triple (H^+^/O^2−^/e^−^) Conducting Nanocomposite of Ba-Co-Ce-Y-O into an Electrolyte for Semiconductor Ionic Fuel Cells

**DOI:** 10.3390/nano11092365

**Published:** 2021-09-11

**Authors:** Dan Xu, An Yan, Shifeng Xu, Yongjun Zhou, Shu Yang, Rongyu Zhang, Xu Yang, Yuzheng Lu

**Affiliations:** 1 College of Science, Shenyang Aerospace University, Shenyang 110136, China; xudan@sau.edu.cn (D.X.); an_137@163.com (A.Y.); zhouyj999@126.com (Y.Z.); yangshu@stu.sau.edu.cn (S.Y.); zhangrongyu1987@foxmail.com (R.Z.); xuyangmark@foxmail.com (X.Y.); 2 Liaoning General Aviation Academy, Shenyang 110136, China; 3 School of Electronic Engineering, Nanjing Xiaozhuang University, Nanjing 211171, China

**Keywords:** twin-perovskite, triple conducting oxides, heterostructure, electrochemical performance

## Abstract

Triple (H^+^/O^2−^/e^−^) conducting oxides (TCOs) have been extensively investigated as the most promising cathode materials for solid oxide fuel cells (SOFCs) because of their excellent catalytic activity for oxygen reduction reaction (ORR) and fast proton transport. However, here we report a stable twin-perovskite nanocomposite Ba-Co-Ce-Y-O (BCCY) with triple conducting properties as a conducting accelerator in semiconductor ionic fuel cells (SIFCs) electrolytes. Self-assembled BCCY nanocomposite is prepared through a complexing sol–gel process. The composite consists of a cubic perovskite (Pm-3m) phase of BaCo_0.9_Ce_0.01_Y_0.09_O_3-δ_ and a rhombohedral perovskite (R-3c) phase of BaCe_0.78_Y_0.22_O_3-δ_. A new semiconducting–ionic conducting composite electrolyte is prepared for SIFCs by the combination of BCCY and CeO_2_ (BCCY-CeO_2_). The fuel cell with the prepared electrolyte (400 μm in thickness) can deliver a remarkable peak power density of 1140 mW·cm^−2^ with a high open circuit voltage (OCV) of 1.15 V at 550 °C. The interface band energy alignment is employed to explain the suppression of electronic conduction in the electrolyte. The hybrid H^+^/O^2−^ ions transport along the surfaces or grain boundaries is identified as a new way of ion conduction. The comprehensive analysis of the electrochemical properties indicates that BCCY can be applied in electrolyte, and has shown tremendous potential to improve ionic conductivity and electrochemical performance.

## 1. Introduction

Solid oxide fuel cells (SOFCs) are extensively studied by both research and industrial communities due in large part to their excellent Faraday efficiency, low greenhouse gases emission and fuel flexibility [1,2,3,4]. Traditional SOFCs consist of a dense oxygen ion conducting electrolyte such as yttria-stabilized zirconia (YSZ) sandwiched between the anode and the cathode. Unfortunately, most oxygen-ion conductors are high-temperature dependent electrolytes, which can only reach the sufficient ionic conductivity (0.1 S cm^−1^) at over 700 °C [5]. High temperature operation results in high cost, performance degradation, long start-up and shut-down cycles, and thus hinders the commercialization of SOFCs [6,7,8]. A decrease in operating temperature to 600 °C or even lower while maintaining considerable electrochemical performance is an imperative need for commercialization of SOFCs technology. Extensive efforts have been devoted to lower the operating temperature of SOFCs based on the typical anode/dense electrolyte/cathode structure. The reduction in the electrolyte thickness and the development of new materials are the two main methods that have been employed. However, the sophisticated technological process and poor mechanical properties impede the cost reduction and the wide implementation as well.

Tremendous efforts have been dedicated to reduce the production cost and promote the performance of the cell based on functional materials and the revolutionary design of the structure of SOFCs [9,10,11,12,13,14,15,16,17]. In recent years, SOFCs based on a ceramic proton conductor–also known as protonic ceramic fuel cells (PCFCs)–have demonstrated outstanding performance. Mobile protons which are found in oxides with perovskite-type structure ABO_3_ with Ba, Ca or Sr on the A-site and Ce, Co or Zr on the B-site have been introduced into both of the electrolyte and cathode systems of SOFCs [18,19,20,21,22]. The low activation energy for proton conduction compared to oxygen-ion conductors and no dilution of fuel during operation are the two main advantages of PCFCs. Small protons can be incorporated at the anode through forming covalent bonds. They can transfer through the dense electrolyte to the cathode by a Grottuss-type mechanism from one ion to the other. The easy proton migration makes ceramic proton conductors very attractive materials for SOFCs operating at reduced temperatures. For instance, it has been reported that a typical proton electrolyte BaCe_0.7_Zr_0.1_Y_0.1_Yb_0.1_O_3-δ_ with a triple charge (H^+^/O^2−^/e^−^) conducting cathode BaCo_0.4_Fe_0.4_Zr_0.1_Y_0.1_O_3-δ_ showed a considerable power density of 445 mW·cm^−2^ at 500 °C and operation was possible even at 350 °C [22]. Kiho et al. fabricated a fuel cell containing thin BaCe_0.5_Zr_0.35_Y_0.15_O_3-δ_ electrolyte which showed a promising power density of 635 mW·cm^−2^ at 600 °C, with reasonable OCV of over 1 V [23]. Besides, BaFeO_3-δ_ based perovskite materials such as BaCe_x_Fe_1-x_O_3-δ_ [24], BaFe_0.95_Sn_0.05_O_3-δ_ [25] and BaZrO_3_ based perovskite materials doped with Co, Fe or Y such as BaZr_1__-x_Co_x_O_3__-δ_ [26], BaCo_0.4_Fe_0.4_Zr_0.1_Y_0.1_O_3-δ_ [27] have been considered as the state-of-the-art electrode materials for SOFCs. These materials may exhibit triple (H^+^/O^2−^/e^−^) conducting behavior, thus improve the cathode performance greatly [28,29]. In comparison with mixed ionic and electronic conducting (MIEC) cathodes, these perovskite materials largely extend the electrochemical reaction active sites for ORR from the limited triple-phase boundary (TPB) to the whole electrode. Song et al. developed an oxygen ion–proton–electron-conducting nanocomposite, BaCo_0.7_(Ce_0.8_Y_0.2_)_0.3_O_3-δ_, which was prepared by one-pot sol–gel method, as a high-performance PCFCs cathode. The nanocomposite was a mixture of H^+^/e^−^ conducting BaCe_x_Y_y_Co_z_O_3-δ_ phase and O^2−^/e^−^ conducting BaCo_x_Ce_y_Y_z_O_3-δ_ and BaCoO_3-δ_ phases [30]. The interfaces between these phases tremendously increased the number of active sites, and thus promoted ORR kinetics of this composite cathode. A high power density of 985 mW·cm^−2^ was achieved at 650 °C when this triple conducting composite cathode was applied in PCFCs [30]. Despite the above significant advantages, there are still some drawbacks of proton conductors. As the electrolyte materials, doped barium zirconate require sintering temperatures over 1600 °C because of their poor sinterability. The high temperature sintering process facilitates BaO evaporation from the lattice which would cause deviation of the stoichiometry of the perovskite and significantly decrease total proton conductivity of the electrolyte [31,32]. The practical applications of cobalt-containing electrode materials also face some problems, such as high price of cobalt, easy evaporation, reduction in cobalt and high thermal expansion coefficient associated with the valence and spin state transition of cobalt ions [33,34].

At the same time, the new architectures in heterostructure materials with a semiconductor and an ionic conductor yield interesting properties with ionic conduction highways between two-phase interfaces [11,12,13,14,15,16,17]. A notable example is CeO_2_/CeO_2-δ_ core-shell structure, which exhibits a super proton conductivity of 0.16 S cm^−1^ for the electrolyte at 520 °C [11]. Unique surface-constrained shuttles for fast proton transport had been built at the interface of the CeO_2_/CeO_2-δ_ heterostructure [11]. Similarly, Naveed reported a heterostructure built by a new redox stable semiconductor SrFe_0.75_Ti_0.25_O_3-δ_ (SFT) and an oxygen-ion conductor Sm_0.25_Ce_0.75_O_2_ (SDC). The materials exhibited a high ionic conductivity > 0.1 S cm^−1^ at 520 °C, which was one order of magnitude higher than that of bulk SDC. The composite as a mixture of ionic and electronic conductors did not suffer the electronic short-circuiting problem. The energy band alignment and heterojunction helped to prevent electrons from passing through the electrolyte. Besides, they believed that electron states modulate local electrical fields to facilitate ion transport [12]. Dong et al. designed a novel fuel cell device from the perspective of the energy band structure and alignment [17]. They fabricated a TiO_2_ thin film to play the role of an electrolyte with sufficient ionic transportation without the electronic short-circuiting problem. An OCV of 1.1 V and maximum power density of 364 mW·cm^−2^ at 550 °C were achieved. More importantly, in contrast to conventional SOFCs, all these heterostructures can present excellent fuel cell performance without the high temperature densification process of the electrolyte, which can effectively avoid the aggravated technological complexity and the expensive fabrication cost. The application of semiconductor materials in the electrolyte provides a new pathway to reducing the operating temperature of SOFCs to an even lower level. Based on different mechanisms, this new kind of SOFC is also called a semiconductor ionic fuel cells (SIFCs) [35,36].

Inspired by the aforementioned ceramic proton conductors and the new design of heterostructure semiconductor electrolytes, here we proposed a novel type of triple-conducting electrolyte system comprised of BCCY and CeO_2_ nanocomposite for SIFCs. The primary objective of this work was to introduce TCOs into undoped CeO_2_ to investigate the potential ability of TCOs in accelerating the conduction of both H^+^ and O^2−^ ions along the interfaces and grain boundaries. A complexing sol–gel process was used to form BCCY nanocomposite. X-ray diffraction measurement and a scanning electron microscope (SEM) were employed to present a comprehensive picture of the crystal structure and microstructure of the investigated material system. The assessment of electrical characterization of the triple-conducting composite electrolyte was carried out by electrochemical impedance spectrum (EIS). The electrochemical performances using the BCCY-CeO_2_ and CeO_2_ as electrolyte, respectively, were also investigated and compared.

## 2. Materials and Methods

### 2.1. Material Preparation

The BCCY nanocomposite, with a nominal composition of BaCo_0.7_Ce_0.24_Y_0.06_O_3-δ_, was synthesized by a sol–gel process of EDTA-citric acid complexing method. Stoichiometric amounts of chemicals C_4_H_6_O_4_Ba, Co(NO_3_)_2_·6H_2_O, Ce(NO_3_)_3_·6H_2_O and Y(NO_3_)_3_·6H_2_O (all of the starting chemicals are from Shanghai Makclin Biochemical Co., Ltd., Shanghai, China) were dissolved in deionized water using EDTA acid and citric acid as complexing agents with heating and continuous stirring at 60 °C. EDTA and citric acid were added to the aqueous solution with the molar ratio of EDTA:citric acid:total metal ions being 1.5:1.5:1. The pH value of the solution was adjusted to approximately 8 by using ammonium hydroxide and nitric acid. After heating and stirring about 5–6 h, the dark purple gel was obtained. The gel was dried in drying case at 150 °C for 24 h to remove the impregnated water and finally calcinated at 1050 °C in air for 10 h to form the primary powder.

On getting the triple-conducting composite electrolyte BCCY-CeO_2_, the prepared BCCY powder was mixed with a commercial purchase CeO_2_ in various mass ratios of 1:1, 1:2, 1:4 and 1:5 followed by thorough blending and grinding in agate mortar. Finally, homogenous composite powder was ready for characterizations and cell fabrication. The obtained samples were labeled as BCCY-1CeO_2_, BCCY-2CeO_2_, BCCY-4CeO_2_ and BCCY-5CeO_2_, respectively.

### 2.2. Fuel Cell Fabrication

The as-prepared BCCY-CeO_2_ and CeO_2_ were employed as the electrolyte for the fuel cell, which were co-pressed between commercially purchased Ni_0.8_Co_0.15_Al_0.05_LiO_2−δ_ (NCAL) (Tianjin Bamo Science and Technology Joint Stock Limited Tianjin, Tianjin, China) painted Ni-foam layers to fabricate symmetrical fuel cell disk. During the fuel cell performance test, the Ni-foam layers with NCAL acted as the anode or cathode according to the gas atmosphere of the respective side of the fuel cell. The dimension of the fabricated fuel cell disk was about 1.0 mm in thickness, 13 mm in diameter and 0.64 cm^2^ in the active area of the electrode, respectively. Afterward, an additional heat-treatment process for the fabricated fuel cell was carried out by sintering 550 °C for 1 h before the performance test.

### 2.3. Characterizations and Electrochemical Measurements

The diffraction patterns of prepared materials were collected by using an X-ray diffractometer (Rigaku Ultima IV diffractometer, 3 kW, Japanese Rigaku Corp., Tokyo, Japan) with Cu Kα radiation in the range of 20° ≤ 2θ ≤ 120°. Furthermore, the structural Rietveld refinement of BCCY was carried out by using FullProf software to obtain structural and profile parameters [37]. To determine the stability of the nanocomposite system under the operating condition of SIFCs, high-temperature X-ray powder diffraction (HT-XRD) of BCCY was carried out using a Rigaku SmartLab SE( Japanese Rigaku Corp., Tokyo, Japan). High-temperature experiments were performed using Anton Paar XRK900. HT-XRD data were collected at 450, 500 and 550 °C in air. The XRD patterns of the BCCY-CeO_2_ were also taken after the heat treatment of the samples under H_2_ and air atmosphere, respectively. The morphology of the fabricated SIFCs was examined using scanning electron microscopy (SEM, Carl Zeiss Sigma and Sigma 300 equipped with Smartedx energy dispersive spectrometer, Carl Zeiss AG, Germany) after the operation of fuel cell performance tests.

The performance characteristics of the fabricated SIFCs were evaluated in a low temperature range of 350–550 °C using a programmable electronic load instrument (IT8511, ITECH Electrical Co., Ltd., Nanjing, China) with hydrogen as fuel gas and air as the oxidant. The flow rate of hydrogen and air were about 120 mL min^−1^ and 150 mL min^−1^, respectively. The electrochemical impedance spectroscopy (EIS) was conducted in the same gas atmosphere as above using CHI660E (CH Instruments Inc, China) under open-circuit potentials (OCP) in a frequency range of 0.1 Hz to 0.1 MHz.

Hall effect measurements were performed at 400 °C using Lake Shore 8400 Series (Lake Shore Cryotronics, Inc. Columbus, Columbus, OH, USA)under an applied current of 2 mA and a 0.5 T magnetic field.

Ultraviolet photoelectron spectroscopy (UPS) was carried out on PHI5000 VersaProbe III (Ulvac-Phi. Inc., Kanagawa, Japan)with an He-I (21.2 eV) discharge lamp to obtain the valence band level. The UV vis-diffused reflection spectra were tested on a Shimadzu UV-3600 spectrophotometer (Shimadzu Corp., Kyoto, Japan)to determine the optical energy bandgap of the materials.

## 3. Results

### 3.1. Material Characterizations

**Figure 1:**
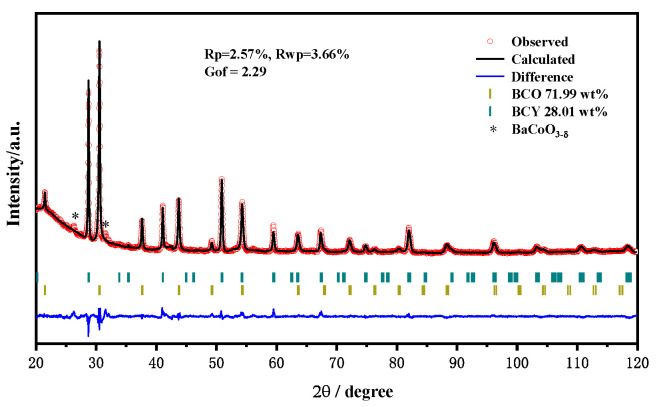
XRD pattern with Rietveld refinement analysis of the BCCY powder.

The room-temperature XRD pattern of BCCY powder and the Rietveld refinement of the pattern are presented in Figure 1. It can be seen that a dual phase system was formed spontaneously. A mixture of BaCo_0.9_Ce_0.01_Y_0.09_O_3-δ_ (BCO), 71.99 wt% and BaCe_0.78_Y_0.22_O_3-__δ_ (BCY), 28.01 wt% was confirmed by Rietveld refinement. Both phases were well crystallized and can be assigned to a typical cubic perovskite structure with space group Pm-3m and a rhombohedral perovskite structure with space group R-3c, which is similar to the data obtained by Song [30]. The refined phase composition and the calculated lattice parameters of BCCY are listed in Table 1. Another two additional peaks located at 26° and 31° are observed, indicating a small amount of BaCoO_3-__δ_ phase.

**Figure 2:**
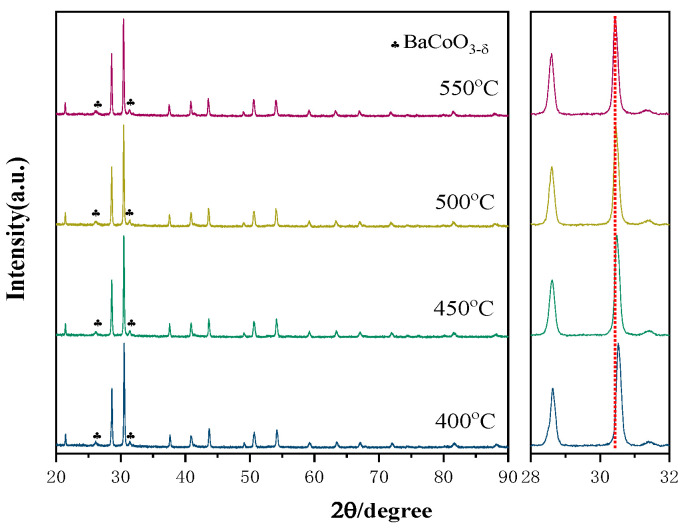
HT-XRD patterns of the BCCY sample from 400 to 550 °C.

**Figure 3:**
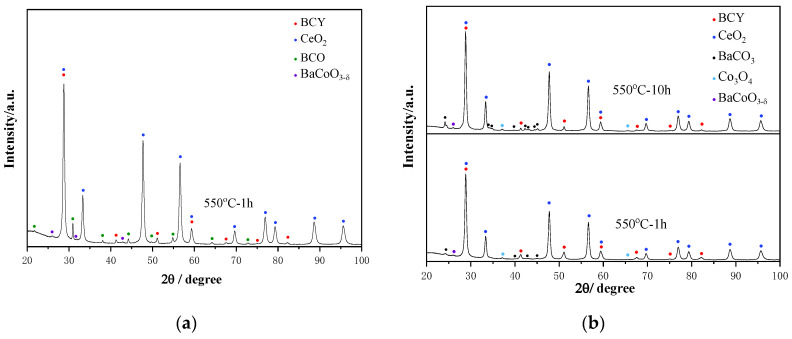
XRD patterns of BCCY-4CeO_2_ after the heat treatment of the sample under (**a**) air and (**b**) H_2_ atmosphere, respectively.

The structural stability is a big concern toward the operation of the fuel cell. Because all the components of the fuel cell have to experience an elevated temperature environment, sufficient structural stability of the electrolyte under the operation conditions is critical to maintain a stable performance of the fuel cell. To explore the structural stability of BCCY under fuel cell operating conditions, high-temperature XRD measurement was performed in air and the patterns are displayed in Figure 2. The XRD patterns of the BCCY show that the composite remained in its twin-perovskite structure over the entire testing temperature range, suggesting high thermal stability of the BCCY composite. The characteristic diffraction peaks shifted to lower two theta as the temperature was increased, which indicates an expansion of the lattice due to thermal reduction in B-site cation in the BCO phase [38]. XRD analysis was also performed to investigate the structural stability of BCCY-CeO_2_ composite after the heat treatment of the samples under a different gas atmosphere. As shown in Figure 3, after the heat treatment of the samples under air for 1 h (Figure 3a), the diffraction peaks consisted of three main phases that can be assigned to individual CeO_2_, BCY and BCO, while after the heat treatment of the samples under H_2_ for 1 and 10 h (Figure 3b), a particular phenomenon could be observed. CeO_2_ and BCY remained in the composite as the major components, but the phase of BCO disappeared. At the same time, Co_3_O_4_ was formed accordingly. Additionally, trace amounts of BaCO_3_ were determined in the composite after 1 h hydrogen treatment, which was caused by the decomposition of BCY. It can also be observed that the diffraction peaks of BaCO_3_ became stronger with the increase in the heat treatment time. The decomposition under reduction atmosphere was consistent with a similar observation reported by Frontera et al. [39]. The result indicates that the electrolyte will present different phase components from the side close to the cathode to the side close to the anode in the condition of the real operation of the fuel cell.

**Figure 4:**
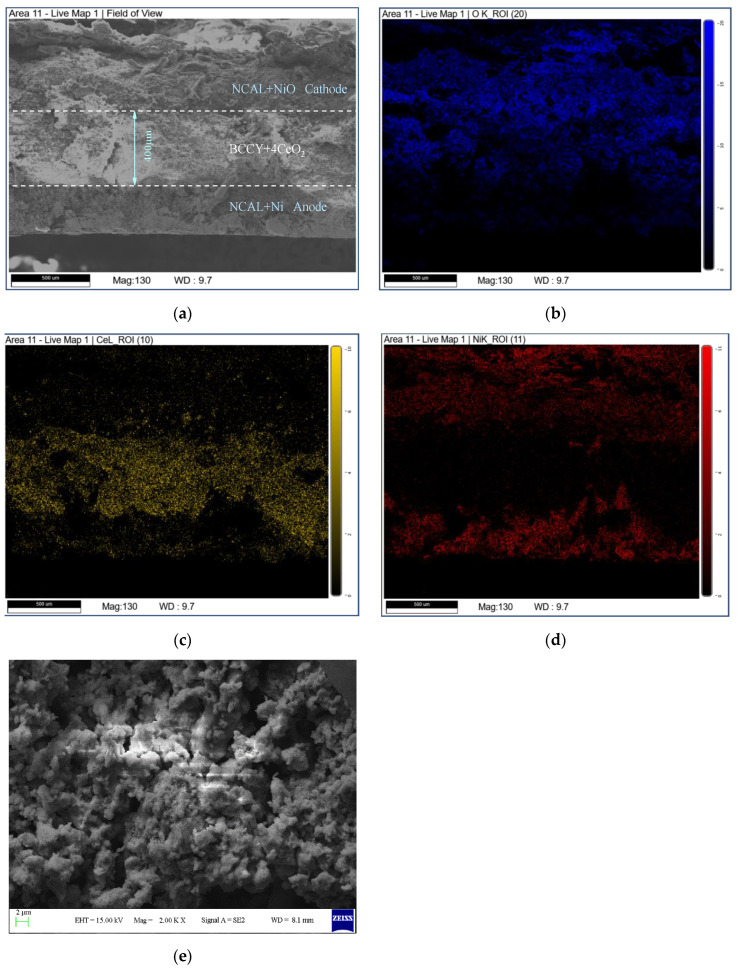
(**a**) Cross-sectional SEM morphology of the BCCY-4CeO_2_ cell after performance test, (**b**–**d**) the corresponding elemental mapping results for O, Ce and Ni, respectively. (**e**) The magnified image of the BCCY-4CeO_2_ surface.

Figure 4 shows the cross section of the tested cell with BCCY-4CeO_2_ as the electrolyte. From Figure 4a, combined with the elemental mapping results, the three layers of the fuel cell can be clearly observed. The electrolyte with the thickness of about 400 μm was firmly sandwiched between the two electrodes after the performance test. Different from the conventional SOFCs, the electrolyte in SIFCs did not undergo high temperature sintering. It can be found in Figure 4e that the electrolyte was not fully dense and some enclosed pores were observed. However, it still could attain excellent electrochemical performance compared with conventional SOFCs. The detail will be discussed later in the series.

### 3.2. Electrochemical Performance and Electrical Properties

**Figure 5:**
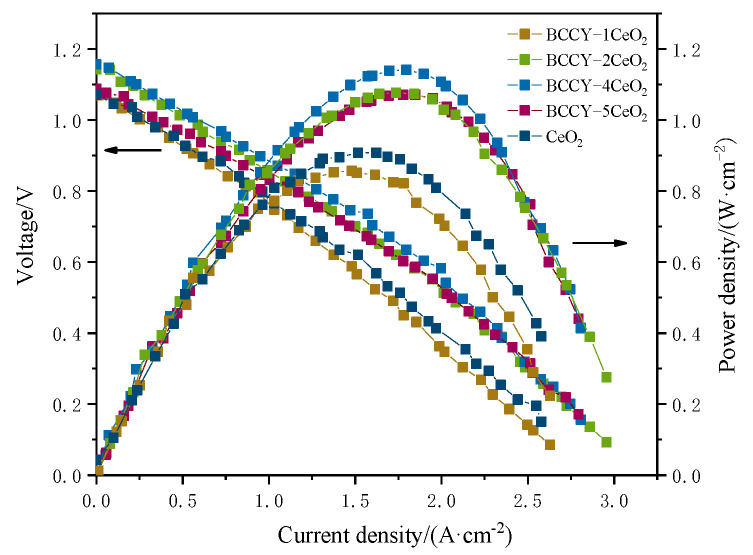
The electrochemical performances for fuel cells using BCCY-CeO_2_ composite with different mass ratios at 550 °C.

**Figure 6:**
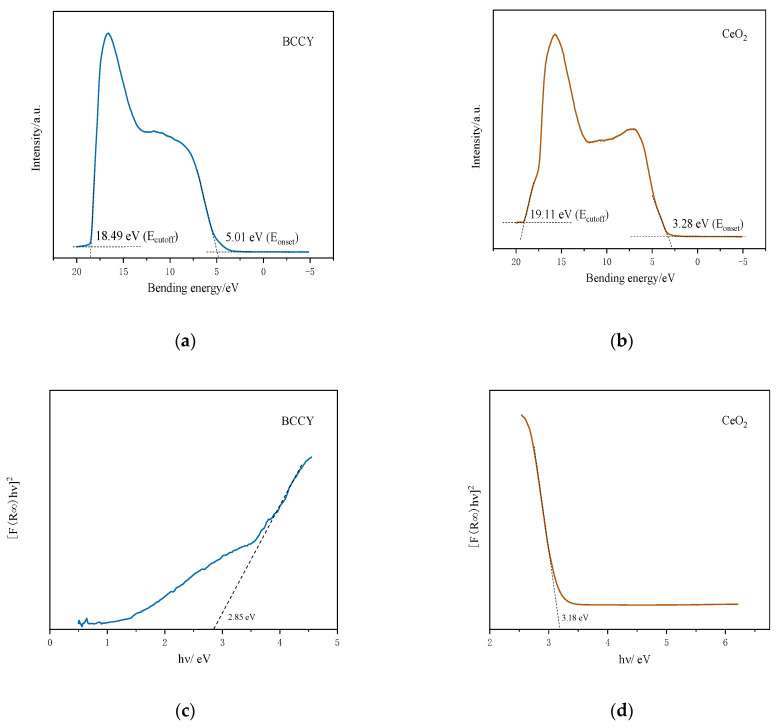
(**a**,**b**) The UPS and (**c**,**d**) the UV vis-diffused reflection spectra of BCCY and CeO_2_.

**Figure 7:**
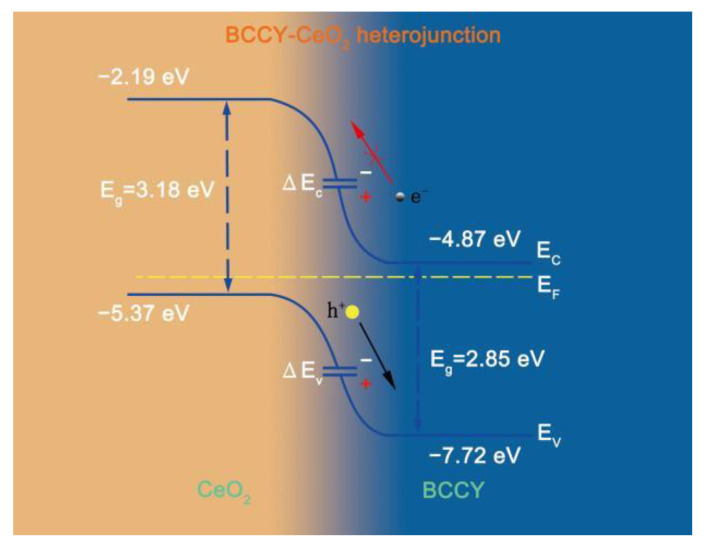
The energy band structure and alignment diagram at the interface of CeO_2_ and BCCY particles.

Figure 5 shows electrochemical performances for cells using BCCY-CeO_2_ composite with different mass ratios at 550 °C. For sake of comparison, the electrochemical performance for CeO_2_ based fuel cell is also presented in the figure. The OCVs for all five cells were higher than 1.05 V. In addition, the OCVs of the fuel cells using BCCY-4CeO_2_ electrolyte at different temperature remained almost unchanged (as shown in Figure S1). All these values are close to Nernst theoretical potential at this temperature [40,41], indicating the satisfactory gas tightness and negligible electronic leakage. In this study, cobalt-containing perovskite oxide, an effective cathode material for conventional SOFCs due to their excellent electrocatalytic activity toward ORR and high electrical conductivity, was designed to be used as the electrolyte in SIFCs. Actually, cobalt-containing perovskite oxide cannot be used as the electrolyte by itself, because of the huge electronic conductivity [22]. However, it is worth noting that no electronic short circuit issue induced by the electronic conduction of BCCY was observed. On the contrary, the appropriate introduction of these triple conducting nanocomposite BCCY can somehow increase the OCV of the SIFCs. The formation of *p*-*n* heterojunction and the effect of band energy alignment between the BCCY and CeO_2_ was proposed to interpret the carrier transportation behavior and barrier to suppress the electron from passing through the electrolyte [17,42]. As reported by Harumi et al., CeO_2_ exhibited *p*-type electronic conduction in the air [43]. In order to ascertain the semiconductor type of BCCY, Hall-effect measurement was performed to examine the carrier type, carrier concentration, hall coefficient and the mobility of the carriers. The parameters are listed in Supplementary Table S1. The result shows that BCCY is *n*-type semiconductor in air circumstance. When these two different types of semiconductors are contacted, the space-charge region with built-in electric field pointing from *n*-type towards the *p*-type region can be established through the redistribution of charges at the interface of these two kinds of materials [44]. The valence band (VB) maxima and energy band level of BCCY and CeO_2_ were obtained by UPS and UV-vis diffused reflection. In the UPS spectra presented in Figure 6a,b, the valence band maxima was determined via defining the cutoff and onset energy. According to E_v_= −[21.2 eV − (E_cutoff_ − E_onset_)] [45], where E_v_ is the VB maxima, E_cutoff_ is the cutoff energy and E_onset_ is the onset energy from UPS, the VB maxima of BCCY and CeO_2_ are calculated to be −7.72 and −5.37 eV, respectively. Based on UV-vis diffused reflection in Figure 6c,d, the bandgap can be determined to be 2.85 eV for BCCY and 3.18 eV for CeO_2_. The conduction band (CB) levels are obtained as −4.87 eV for BCCY and −2.19 eV for CeO_2_, by subtracting bandgap from VB levels. These parameters indicate that BCCY and CeO_2_ can form the desirable *p*-*n* heterojunction in the electrolyte. Figure 7 illustrates the energy band structure and alignment by band bending at the interface of the BCCY and CeO_2_ particles. Due to the different energy band levels of BCCY and CeO_2_, the conduction band offset (ΔE_c_) and the valence band offset (ΔE_v_) will adjust to reach a continuous Fermi energy level throughout the whole interface. Potential barriers and built-in electric field in this region will impede transfer of the generated and intrinsic electrons from BCCY to CeO_2_, thus preventing the electronic conduction from the anode side to the cathode side through electrolyte.

**Figure 8:**
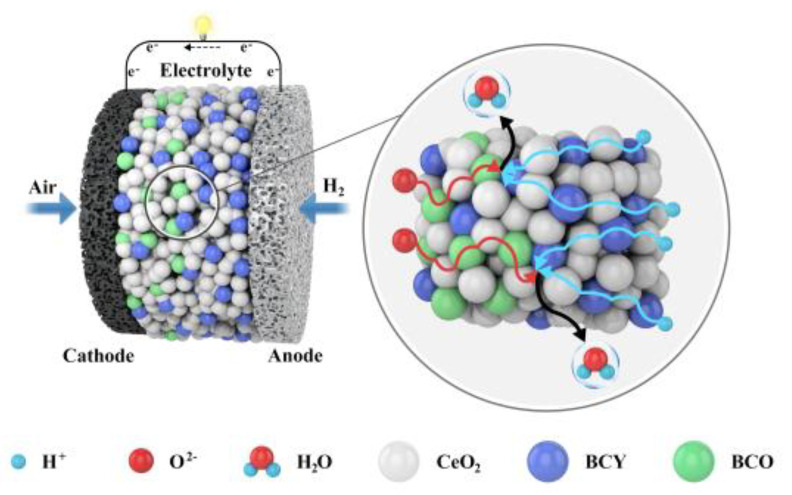
Schematic illustration for the fuel cell using BCCY-CeO_2_ composite as the electrolyte and proposed transport mechanism for H^+^ and O^2−^ ions.

Another notable characteristic that can be seen in the Figure 5 is the peak power density of the cells fabricated with BCCY-CeO_2_ composite electrolytes are much higher than that of the SIFCs using CeO_2_ as electrolyte. The peak power density increases with the increase in the amount of CeO_2_ and then drops with the further increase in CeO_2_ content. Remarkable peak power density of 1140 mW·cm^−2^ is obtained for BCCY-4CeO_2_, which is even higher than those of the conventional SOFCs using condensed YSZ, doped-CeO_2_ electrolytes under the identical operating conditions [46,47]. High performance of the cell is a result of the triple (H^+^/O^2−^/e^−^) conducting nature of perovskite BCCY. Figure 8 shows schematic illustration for the fuel cell using BCCY-4CeO_2_ as the electrolyte and proposed transport mechanism for H^+^ and O^2−^ ions. When hydrogen and air are supplied to each side of the cell, corresponding electrode reactions take place. The elementary reaction steps on the cathode side can be described as:

O_2(g)_ → O_2ads_ (oxygen adsorption) (1)

O_2ads_ → 2O_ads_ (oxygen dissociation) (2)

O_ads_ + 2e^−^ → O^2−^_ads_ (oxygen reduction) (3)

On the anode side, the elementary reactions follow the steps as:

H_2(g)_ → H_2ads_ (hydrogen adsorption) (4)

H_2ads_ → 2H_ads_ (hydrogen dissociation) (5)

H_ads_ → H^+^_ads_+ e^−^ (hydrogen oxidation) (6)

On both side of the cell, all the gas molecules from the surrounding atmosphere undergo the adsorption and dissociation process to form O^2−^ or H^+^ ions. The O^2−^ or H^+^ ions must migrate through the entire thickness of the electrolyte, when the electrolyte presents single particle conduction. Finally, the ions arrive at the other side the cell until encountering H^+^ or O^2−^ to form H_2_O. Electricity generation is completed in the process.

According to the XRD results of this study, the dual phases of the BCCY composite could automatically redistribute in the electrolyte layer of the cell. BCY, as one of the doped barium cerates, which presents mixed proton and electronic conductivity [48], can be found in the whole area of the electrolyte, but the content reduces from the anode side to the cathode side. Meanwhile BCO as one of the barium cobalt oxides which presents mixed oxygen ion and electronic conductivity [49] assembles near to the cathode side. As shown in Figure 8, a double layer configuration of the electrolyte can be formed spontaneously. Additionally, the reduction of the Ce^4+^ to Ce^3+^ in the reducing atmosphere can provide a shuttle for the transportation of protons along the surface of CeO_2_ particles [11]. Ce^4+^ can be balanced by Ce^3+^ and H^+^ to maintain the charge compensation for neutrality without the formation of the conventional oxygen vacancy [50]. The corresponding transport paths of the ions can be shortened to at least half of the original length. The formation of H_2_O occurs as long as H^+^ and O^2−^ appear in close proximity. Different from the conventional SOFC, all reactions and processes are completed on the surfaces of the particles through direct combination of H^+^ and O^2−^ ions.

**Figure 9:**
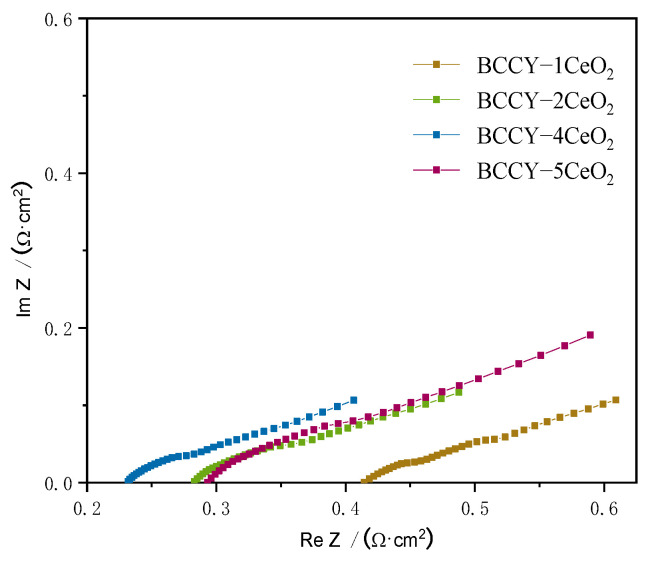
Electrochemical impedance spectroscopy of the fuel cell with BCCY-CeO_2_ composite electrolyte at 450 °C.

Figure 9 shows the electrochemical impedance spectroscopy of the fuel cells with the BCCY-CeO_2_ composite electrolytes at 450 °C. The intercept on the real axis at high-frequency represents the ohmic resistance (R_o_), which mainly originates from the electrolyte. As shown in Figure 9, the ohmic area-specific resistances (ASRs) are 0.415 Ω cm^2^, 0.283 Ω cm^2^, 0.231 Ω cm^2^ and 0.294 Ω cm^2^ for the cells with BCCY-*x*CeO_2_ composite electrolyte (*x* = 1, 2, 4 and 5, respectively). The fuel cell of BCCY-4CeO_2_ presents the lowest ohmic ASR among all the cells. The electrochemical impedance spectroscopy of the fuel cell with BCCY-4CeO_2_ electrolyte at different temperature are shown in Figure S2. Compared to the typical conventional SOFCs with a single proton conducting or oxide-ion conducting electrolyte, our fuel cell showed similar ohmic ASRs, but significantly higher performances vs the references (as shown in Table S2). Generally, the intercept on the X-axis at high frequencies of the impedance spectra represents bulk resistance of the electrolyte [51]. Bulk conduction and diffusion is the primary ion migration mechanism for conventional dense ceramic electrolytes [52,53]. From the cross-sectional SEM morphology of the fuel cell shown in Figure 4a, the condition is quite different in the present work. Several researchers have reported that the formation of a *p*-*n* heterojunction between the oxide ion conductor and the semiconductor in a composite can significantly promote the ionic conductivity of the electrolyte [54]. Naveed Mushtaq et al. developed a novel Ba_0.5_Sr_0.5_Fe_0.8_Sb_0.2_O_3-δ_-Sm_0.2_Ce_0.8_O_2-δ_ (BSFSb-SDC) heterostructure. They found that the heterostructure provided a faster charge transfer and enhanced ion transport through interface. The built-in electric field induced by the unbalanced charge distribution between the Ce–O and Fe–O layers assisted the high ionic conductivity at the interface of BSFSb and SDC [12]. Similarly, the built-in electric field formed between BCCY and CeO_2_ particles in this study facilitated the ionic migration through the heterointerface regions, thus enhancing the BCCY-CeO_2_ fuel cells performance.

It should be noted that longer-term durability is a crucial aspect of the performance of the fuel cell. As mentioned above, after the long-time treatment of the electrolyte under hydrogen atmosphere, BaCO_3_ was formed. Until now, we have not seen any negative impact of BaCO_3_ on the performance of the fuel cell. Tao Hong et al. reported that BaCO_3_ can improve the chemical oxygen surface exchange coefficients by a large factor [55]. In the present work, electrode reactions took place throughout the entire area of the device. The synergistic effect of BaCO_3_ in this new SIFCs system is worth investigating. Extensive research should be undertaken on the role of the byproduct in the device and on the engineering efforts to enhance the device stability in the future.

## 4. Conclusions

In summary, BCCY, which possesses a dual phase by self-assembly process, was developed as a novel triple (H^+^/O^2^^−^/e^−^) conducting electrolyte material for low-temperature SIFCs. The material was successfully fabricated through a complexing sol–gel method. A heterostructure was established by the combination of an appropriate amount of BCCY with CeO_2_. Low-temperature SIFCs was prepared through the simplest process of pressing all the components of the cell together without high temperature sintering of the electrolyte. The peak power density with BCCY-4CeO_2_ achieved 1140 mW·cm^−2^ with a high OCV of 1.15 V in H_2_/air at 550 °C. Structural characterization shows that the dual phase of BCO and BCY in BCCY can redistribute under the fuel cell operating condition, which could promote the migration of the H^+^ and O^2^^−^ ions. A desirable *p*-*n* heterojunction formed between BCCY and CeO_2_ particles could help to prevent the electronic conduction induced by BCCY. Additionally, the built-in electric field between BCCY and CeO_2_ could facilitate the ionic migration through the heterointerface regions, thus enhancing the BCCY-CeO_2_ fuel cell performance. It is demonstrated that the TCOs are potential electrolytes for SIFCs, which can dramatically increase the power outputs of the fuel cell. This work presents a new rational way to design the electrolyte of SIFCs.

## Figures and Tables

**Table 1:** The refined phase composition and the calculated lattice parameters of BCCY.

Phase	a (Å)	b (Å)	c (Å)	Volume (Å^3^)
BaCe_0.78_Y_0.22_O_3-δ_	6.21239 (14)	6.21239 (14)	15.2452 (7)	509.543 (3)
BaCo_0.9_Ce_0.01_Y_0.09_O_3-δ_	4.13906 (4)	4.13906 (4)	4.13906 (4)	70.9096 (13)

## Data Availability

Data are contained within the article or Supplementary Materials.

## Supplementary Materials

The following are available online at www.mdpi.com/article/10.3390/nano11092365/s1, Figure S1: The electrochemical performances for fuel cells using BCCY-4CeO_2_ electrolyte at different temperature; Figure S2: Electrochemical impedance spectroscopy of the fuel cell with BCCY-4CeO_2_ electrolyte at different temperature; Table S1: The Hall-effect parameters of BCCY at 400 °C in air circumstance; Table S2: Comparison of the ohmic ASR, peak power density and open circuit voltage (OCV) between the SOFCs with the composite electrolyte this work and the conventional electrolyte.
