# Supplementary material for: Self-Assembled Triple (H+/O2−/e−) Conducting Nanocomposite of Ba-Co-Ce-Y-O into an Electrolyte for Semiconductor Ionic Fuel Cells
Source: Nanomaterials (Basel). 2021 Sep 11;11(9):2365. doi: 10.3390/nano11092365 (PMC8472293; doi:10.3390/nano11092365)
Supplement: Supplementary file 1 [file nanomaterials-11-02365-s001.zip › nanomaterials-1331477-supplementary.pdf]

nanomaterials

Article

Self-Assembled Triple ($H^+/O^{2-}/e^-$) Conducting Nanocomposite of Ba-Co-Ce-Y-O into an Electrolyte for Semiconductor Ionic Fuel Cells

Dan Xu [1], An Yan [1], Shifeng Xu [1,2,*], Yongjun Zhou [1], Shu Yang [1], Rongyu Zhang [1], Xu Yang [1] and Yuzheng Lu [3,*]

1 College of Science, Shenyang Aerospace University, Shenyang 110136, China; xudan@sau.edu.cn (D.X.); 952270756@qq.com (A.Y.); zhouyj999@126.com (Y.Z.); yangshu@stu.sau.edu.cn (S.Y.); zhangrongyu1987@foxmail.com (R.Z.); xuyangmark@foxmail.com (X.Y.)
2 Liaoning General Aviation Academy, Shenyang 110136, China
3 School of Electronic Engineering, Nanjing Xiaozhuang University, Nanjing 211171, China
* Correspondence: sfxu@sau.edu.cn (S.X.); luyuzheng@njxzc.edu.cn (Y.L.)

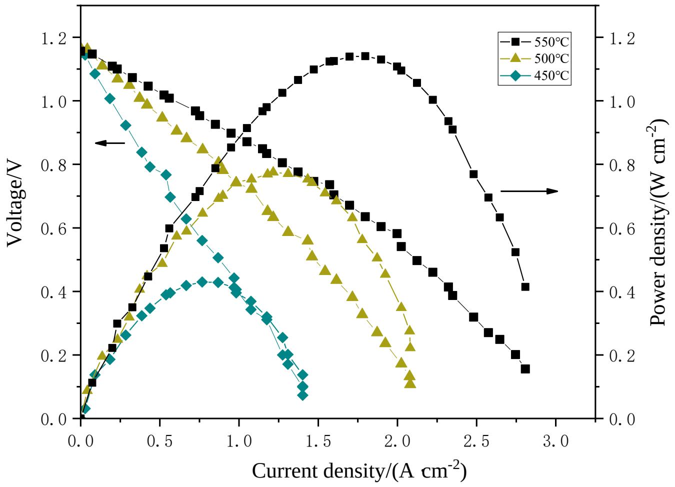

Figure S1. The electrochemical performances for fuel cells using BCCY-4CeO_2 electrolyte at different temperature.

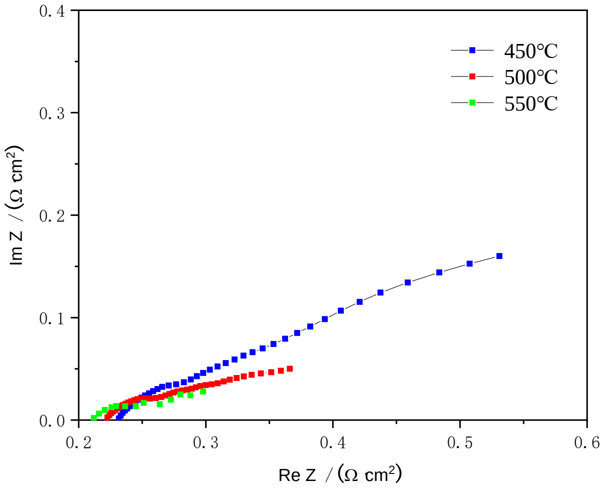

Figure S2. Electrochemical impedance spectroscopy of the fuel cell with BCCY-4CeO_2 electrolyte at different temperature.

Table S1. The Hall-effect parameters of BCCY at 400°C in air circumstance.

Sample	Carrier type	Carrier concentration	Hall coefficient	Mobility of the carrier
BCCY	n	2.28×10^{16} (1/cm^3)	−27930 (cm^3/C)	86.32 (cm^2/V·s)

Table S2. Comparison of the ohmic ASR, peak power density and open circuit voltage (OCV) between the SOFCs with the composite electrolyte this work and the conventional electrolyte.

Electrolyte	ASR(Ω·cm^2)	Peak Power Density(mW/cm^2)	OCV(V)	Ref.
This work	0.21 (550°C)	1140	1.05	This work
$BaCe_{0.7}Zr_{0.1}Y_{0.1}Yb_{0.1}O_{3-\delta}$ −1.0wt.% NiO	0.35 (550°C)	520	1.1	[1]
$La_{0.8}Sr_{0.2}Ga_{0.83}Mg_{0.17}O_{3-\delta}$−La-doped CeO_2	0.21~0.24 (800°C)	1000	1.05	[2]
$BaZr_{0.3}Ce_{0.5}Y_{0.2}O_{3-\delta}$−2 mol% Bi_2O_3	0.23 (650°C)	440	0.99	[3]
$BaZr_{0.3}Ce_{0.5}Y_{0.2}O_{3-\delta}$	0.40 (650°C)	513	1.01	[4]

References

[1] Duan, C.; Tong, J.; Shang, M.; Stefan, N.; Sanders, M.; Ricote, S.; Almansoori, A.; O'Hayre, R. Readily processed protonic ceramic fuel cells with high performance at low temperatures. *Science* **2015**, *349*, 1321-1326. [CrossRef]

[2] Zhu, T.; Troiani, H. E.; Mogni, L. V.; Han, M.; Barnett, A. B. Ni-Substituted Sr (Ti, Fe) O_3 SOFC anodes: Achieving high performance via metal alloy nanoparticle Exsolution. *Joule* **2018**, *2*, 478-496. [CrossRef]

[3] Gui, L.; Ling, Y.;Li, G.; Wang, Z.; Wan, Y.; Wang, R.; He, B.; Zhao, L. Enhanced sinterability and conductivity of $BaZr_{0.3}Ce_{0.5}Y_{0.2}O_{3-\delta}$ by addition of bismuth oxide for proton conducting solid oxide fuel cells. *J. Power Sources* **2016**, *301*, 369-375. [CrossRef]

[4] Shi, Z.; Sun, W.; Liu, W. Synthesis and characterization of $BaZr_{0.3}Ce_{0.5}Y_{0.2-x}Yb_xO_{3-\delta}$ proton conductor for solid oxide fuel cells. *J. Power Sources* **2014**, *245*, 953-957. [CrossRef]
